# Molecular Analysis Based on Fine-Needle Aspiration Washout Samples in Thyroid Nodules

**DOI:** 10.3390/genes17010099

**Published:** 2026-01-19

**Authors:** Sevgül Fakı, Cevdet Aydın, Şefika Burçak Polat, Gülsüm Karahmetli, Ahmet Cevdet Ceylan, Mustafa Altan, Ayşegül Aksoy Altınboğa, Bülent Çomçalı, Oya Topaloğlu, Reyhan Ersoy, Bekir Çakır

**Affiliations:** 1 Department of Endocrinology and Metabolism, Ankara Bilkent City Hospital, Ankara 06800, Türkiye; 2 Department of Endocrinology and Metabolism, Faculty of Medicine, Ankara Yıldırım Beyazıt University, Ankara 06010, Türkiye; 3 Department of Medical Genetics, Faculty of Medicine, Ankara Yıldırım Beyazıt University, Ankara 06010, Türkiye; 4 Department of Pathology, Faculty of Medicine, Ankara Yıldırım Beyazıt University, Ankara 06010, Türkiye; 5 Department of General Surgery, Ankara Bilkent City Hospital, Ankara 06800, Türkiye

**Keywords:** thyroid nodule, indeterminate cytology, Bethesda III–IV, BRAF, RAS, FNA washout, molecular testing, diagnostic accuracy

## Abstract

Background: Molecular testing is recommended to refine risk stratification in indeterminate thyroid nodules (Bethesda III–IV), but data on dual-gene (BRAF and RAS) testing using fresh FNA washout specimens are limited. We aimed to evaluate the performance of BRAF and RAS mutation analysis from fresh thyroid FNA washout material, with a focus on indeterminate cytology. Methods: We retrospectively analyzed 1139 patients who underwent washout-based molecular testing between May 2022 and October 2024 at a tertiary endocrine center. Of these, 307 had available histopathologic results after surgery. Primary outcomes were sample adequacy, mutation spectrum, and diagnostic metrics (sensitivity, specificity, PPV, NPV, and accuracy). Analyses were repeated under two assumptions that classified borderline/low-risk neoplasms as benign vs. malignant, and within the Bethesda III–IV subset. Results: Adequate material for molecular analysis was obtained in 1037/1139 samples (90.9%). In the operated cohort (*n* = 307), malignant lesions comprised 31.9% and low-risk neoplasms 8.5%. When borderline lesions were considered benign, mutation positivity yielded a sensitivity of 48.0%, a specificity of 89.6%, a PPV of 75.9%, an NPV of 71.9%, and an accuracy of 72.9%. In Bethesda III–IV nodules (*n* = 153), sensitivity, specificity, and accuracy were 41.0%, 85.2%, and 66.0% (malignant assumption). Isolated BRAF positivity showed high specificity (~96.7%) with modest sensitivity. Conclusions: Our findings extend current diagnostic approaches by showing that dual-gene (BRAF and RAS) testing from fresh FNA washouts is technically feasible (≥90% adequacy) and provides high specificity with modest sensitivity for malignancy in indeterminate nodules. In settings lacking comprehensive commercial panels, this low-complexity approach offers a practical adjunct to cytology and imaging for preoperative decision-making.

## 1. Introduction

Since the 1980s, the worldwide occurrence of thyroid cancer has shown a remarkable upward trend, while death rates have remained largely unchanged. This discrepancy is thought to stem mainly from the increased detection of small, slow-growing papillary thyroid tumors, a consequence of the expanding and occasionally unnecessary use of diagnostic imaging, particularly high-resolution thyroid and neck ultrasonography. In addition, a modest but genuine rise in the incidence of more advanced forms of thyroid cancer over recent decades has further contributed to the overall increase in reported cases [1].

Fine-needle aspiration (FNA) remains the cornerstone diagnostic technique for evaluating thyroid nodules. Approximately one-third of thyroid fine-needle aspiration biopsies are classified as Bethesda category III or IV, placing clinicians in a challenging position when determining the appropriate management strategy [2]. This category includes Bethesda III (atypia of undetermined significance [AUS]) and Bethesda IV (follicular neoplasm [FN]) classifications. The probability of malignancy within the indeterminate cytology categories varies, but according to the most recent Bethesda System for Reporting Thyroid Cytopathology, it is estimated to be approximately 22% for category III and around 30% for category IV. These risk estimates, however, show substantial variation across different institutions and patient populations. Owing to this diagnostic uncertainty, individuals with Bethesda III or IV nodules are frequently subjected to exploratory thyroid surgery, many of which ultimately prove to be unwarranted once postoperative histology confirms benign pathology [3,4].

Molecular testing is now a key component of preoperative thyroid nodule evaluation, enabling detection of genetic alterations that reflect tumor behavior. Commonly assessed mutations include BRAF, RAS (HRAS, KRAS, and NRAS), TERT promoter, RET/PTC, and TP53. RAS-like mutations usually indicate indolent tumors suitable for hemithyroidectomy, whereas BRAF-like alterations suggest aggressive disease with a higher risk of extrathyroidal spread or nodal metastasis, often warranting total thyroidectomy. Major professional societies now recognize the value of molecular testing for Bethesda III and IV nodules. The 2025 American Thyroid Association (ATA) guidelines recommend its use as an adjunct tool to better define malignancy risk and inform surgical planning [5].

These molecular tests have a high negative predictive value; therefore, the most widely used assays can reliably predict benign nodules that do not require surgical intervention. Moreover, their specificity and positive predictive value have been enhanced through the integration of artificial intelligence and deep learning algorithms, allowing for improved identification of high-risk tumors [6]. Unfortunately, the cost of these tests remains substantial, and they are not currently reimbursed within the national healthcare system in our country.

This study was conducted in Türkiye, where comprehensive commercial molecular testing panels are not routinely reimbursed by the national healthcare system. Although TERT promoter mutations and RET/PTC rearrangements are technically available in our center, these assays are not covered in routine endocrine practice. Therefore, only BRAF and RAS analyses, both of which are reimbursed and provided free of charge, were performed consistently during the study period. We aimed to retrospectively evaluate the sensitivity and specificity of BRAF and RAS mutation analysis performed on fresh washout fluid samples obtained from fine-needle aspiration (FNA) of thyroid nodules, specifically in cases with indeterminate cytology. Since there are only a limited number of studies employing this technique, another primary objective of our research was to assess the feasibility and success rate of DNA extraction from the FNA washout material.

## 2. Materials and Methods

This retrospective study included patients who underwent thyroid fine-needle aspiration (FNA) biopsy between May 2022 and October 2024 at the Endocrinology and Metabolic Diseases Outpatient Clinic. The decision of biopsy was given by endocrinology residents according to the American College of Radiology Thyroid Imaging Reporting and Data System (ACR TI-RADS) criteria [7]. All participants provided written informed consent for the FNA procedure and for their samples to undergo genetic testing. Molecular testing was primarily performed in nodules with indeterminate cytology, particularly Bethesda category III and IV, where genetic analysis was considered most clinically informative. In selected cases, molecular testing was also applied to other cytological categories when additional diagnostic or clinical benefit was anticipated. These included nodules with repeatedly nondiagnostic cytology (Bethesda I), large nodules with benign cytology (Bethesda II), and nodules with suspicious or malignant cytology (Bethesda V or VI) in which molecular analysis was expected to provide supplementary information for clinical decision-making.

Detection of BRAF and RAS mutations was performed using the residual material remaining in the syringe after smear preparation. This residual aspirate was washed with 1 cc of QIAGEN GmbH solution to obtain cellular material for molecular analysis. DNA isolation was performed using the EasyPGX^®^ Extraction Kit (Diatech Pharmacogenetics S.r.l., Jesi, Italy) in accordance with the manufacturer’s instructions. The concentration and quality of the extracted DNA were assessed using a spectrophotometric method; low-yield samples were reprocessed or excluded from the analysis according to quality control criteria. Mutation analyses were carried out using an RT-PCR-based panel. The assay screened for BRAF and RAS gene variants that had been previously reported in the literature and were known to have clinical significance. Positive, negative, and workflow controls were included in each RT-PCR run. Instrument settings, primer/probe sets, and amplification programs were configured according to the manufacturer’s recommendations. A positive result was defined based on the cycle threshold (CT) value specified by the manufacturer or the validation threshold previously established by the laboratory. In comparative analyses, the detection of any BRAF or RAS mutation was considered “molecular marker positive.”

Surgical referral was determined through a multidisciplinary evaluation incorporating cytological, radiological, molecular, and clinical findings. Surgery was primarily recommended for patients with malignant or suspicious cytology (Bethesda V–VI), for indeterminate nodules (Bethesda III–IV) with high-risk ultrasonographic features according to ACR TI-RADS, or in the presence of pathogenic BRAF or RAS mutations suggesting increased malignancy risk. Additional indications for surgery included significant nodule growth during follow-up, compressive symptoms, patient preference, and discordance between cytology and imaging findings. In selected cases with benign cytology (Bethesda II), surgery was considered when nodules were large, symptomatic, or showed suspicious radiological progression.

Nodules with confirmed matching in ultrasonography–cytology–histopathology correlation were evaluated to ensure accurate lesion identification.

Statistical Analysis

Data from a total of 1472 participants were included in the statistical analysis. All analyses were conducted using IBM SPSS Statistics for Windows, version 26.0 (IBM Corp., Armonk, NY, USA). Categorical variables are expressed as frequencies and percentages (n, %), whereas continuous variables are summarized as means ± standard deviations (SD).

The Kolmogorov–Smirnov test was used to assess the normality of distribution for continuous variables, and the results indicated that the data did not follow a normal distribution. Therefore, non-parametric statistical methods were applied for subsequent analyses. Comparisons between two independent groups were performed using the Mann–Whitney U test, while comparisons among more than two groups were evaluated with the Kruskal–Wallis H test. When a significant difference was detected using the Kruskal–Wallis test, Bonferroni-adjusted post hoc pairwise comparisons were employed to identify the source of significance.

Associations between categorical variables were analyzed using the chi-square test, and when the assumption of expected cell frequencies was not met, results from the Likelihood ratio test were reported. A two-tailed *p*-value < 0.05 was considered statistically significant for all analyses.

## 3. Results

In the overall screened cohort (*n* = 1472), a total of 342 patients underwent surgery, while 797 patients were managed conservatively under active surveillance. Additionally, 333 patients were excluded from the analysis, including those who were lost to follow-up (196 patients, 13.3%), those who declined surgery despite recommendation (83 patients, 5.6%), those who were lost to follow-up after surgery was recommended (35 patients, 2.4%), and patients with a pending surgical outcome at the time of analysis (19 patients, 1.3%)

Of the 1472 screened patients, 1139 were eligible and included in the study, with a mean age of 52.2 ± 12.6 years (range: 19–85). The majority were female (82.3%). Nearly half of the patients’ nodules were detected (45.7%) during follow-up for hypothyroidism, hyperthyroidism, hyperparathyroidism, or another endocrinologic comorbid condition. Other reasons for evaluation included incidental findings (8.5%); non-specific complaints (9.7%); neck swelling (5.2%); thyroid-related symptoms such as dysphagia, dyspnea, or pain (5.4%); abnormal antibodies or thyroid function tests (8.1%); and a family history of thyroid cancer (1.1%). In 186 patients (16.3%), the reason for referral was not recorded in the medical records.

In 1139 samples, adequate material for molecular analysis was obtained in most cases. Among the 1139 samples analyzed, 1037 (90.9%) were reported as sufficient for testing (Table 1).

**Figure 1:**
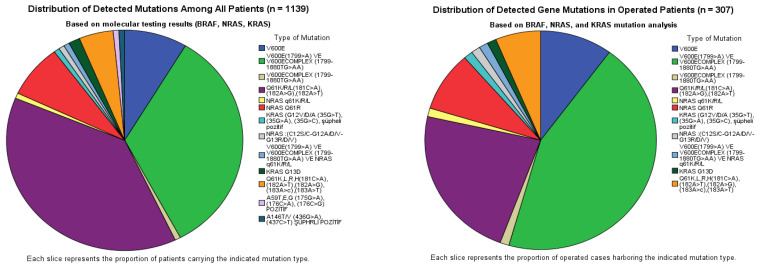
Distribution of detected BRAF, NRAS, and KRAS mutations among all analyzed samples (*n* = 1139) and the operated subset (*n* = 307).

Among the 1139 analyzed thyroid FNA washout samples, 128 (11.2%) were mutation-positive. BRAF mutations were identified in 55 cases (43.0%), predominantly involving the V600E locus, whereas NRAS mutations were detected in 67 cases (52.3%), predominantly involving codon 61. In the operated subset of 307 patients, mutations were detected in 79 cases (25.7%). The overall distribution followed a similar pattern but with a relative enrichment of BRAF mutations (55.7%) compared with NRAS (40.5%), while KRAS remained uncommon (2.5%) (Table 2) (Figure 1). According to Table 2, BRAF mutation-positive cases showed the highest surgical rate, with 44 of 55 patients (80.0%) undergoing surgery. In contrast, NRAS mutation-positive patients were less frequently operated on, with surgery performed in 32 of 67 patients (47.8%). KRAS mutation-positive cases had the lowest surgical rate, with two of five patients (40.0%) undergoing surgery. The single patient harboring a BRAF + NRAS co-mutation underwent surgery (1/1, 100%). The co-mutated patient was a 39-year-old woman with a subcentimetric hypoechoic nodule located in the isthmus. Final pathology revealed multifocal papillary thyroid microcarcinoma, and the dominant lesion showed minimal infiltration into the perithyroidal fat tissue. Overall, 79 of 128 mutation-positive patients (61.7%) were treated surgically. Among mutation-positive patients who did not undergo surgery (*n* = 49), clinical management varied according to risk stratification and patient preference.

Follow-up was planned for 15 patients who were considered low risk based on TI-RADS assessment and/or long-term stable follow-up findings. Surgery was recommended for five patients; however, these patients declined surgical intervention and preferred continued surveillance. In nine patients, surgery was planned, but surgical outcomes were not yet available at the time of analysis. Additionally, 22 patients were lost to follow-up and had no further clinical data available.

The initial fine-needle aspiration biopsy (FNAB) results showed that 83 patients (27.0%) were classified as nondiagnostic (Bethesda I), 15 (4.9%) as benign (Bethesda II), 221 (71.9%) as atypia of undetermined significance/follicular lesion of undetermined significance (Bethesda III), 6 (2.0%) as follicular neoplasm/suspicious for a follicular neoplasm (Bethesda IV), and 2 patients each (0.7%) as suspicious for malignancy (Bethesda V) and malignant (Bethesda VI).

Based on these initial FNAB results, genetic molecular testing was performed, and subsequent reclassification showed that the number of nondiagnostic cases (Bethesda I) remained unchanged at 83 patients (27.1%), while Bethesda II increased to 36 patients (11.8%). In contrast, Bethesda III decreased from 221 patients (71.9%) to 148 patients (48.4%), and Bethesda IV was identified in 4 patients (1.3%). Higher-risk cytological categories increased, with Bethesda V observed in 11 patients (3.6%) and Bethesda VI in 25 patients (8.2%).

A total of 342 patients underwent thyroid surgery; 5 patients were excluded due to discordance between cytological and histopathological findings, and an additional 30 patients were excluded because insufficient or partially sufficient washout material was obtained (18 with only BRAF testing performed, 5 with inadequate material, 3 with only RAS testing performed, 2 with only KRAS testing performed, and 2 with only KRAS testing performed showing normal results), resulting in 307 patients being included in the final statistical analysis.

According to the WHO 2022 classification, histopathological evaluation of 307 cases showed that the majority were benign (*n* = 183, 59.6%), followed by malignant lesions (*n* = 98, 31.9%) and low-risk neoplasms (borderline tumors) (*n* = 26, 8.5%). The borderline tumor group comprised 19 noninvasive follicular thyroid neoplasms with papillary-like nuclear features (NIFTP) and 7 tumors of uncertain malignant potential (UMP). The UMP subgroup included four well-differentiated thyroid tumors of uncertain malignant potential, one follicular tumor of uncertain malignant potential, one well-differentiated follicular neoplasm of uncertain malignant potential, and one neoplasm of uncertain malignant potential, not otherwise specified (NOS).

When borderline lesions were classified as benign, mutation positivity in the overall cohort (*n* = 307) demonstrated a sensitivity of 51.0%, a specificity of 86.1%, a positive predictive value (PPV) of 63.3%, a negative predictive value (NPV) of 78.9%, and an overall diagnostic accuracy of 74.0%. When these borderline lesions were instead considered malignant, sensitivity slightly decreased to 48.0%, whereas specificity increased to 89.6%. Correspondingly, PPV improved to 75.9%, NPV to 71.9%, and overall accuracy remained comparable (72.9%). In the Bethesda III–IV subgroup (*n* = 153), when low-risk neoplasms were categorized as benign, mutation testing yielded a sensitivity of 38.8%, a specificity of 79.8%, a PPV of 47.5%, an NPV of 73.5%, and a diagnostic accuracy of 66.6%. Reclassifying these lesions as malignant resulted in a modest improvement, with sensitivity rising to 41.0%, specificity to 85.2%, PPV to 67.5%, NPV to 66.4%, and an overall accuracy of 66.0% (Table 3).

Isolated BRAF positivity demonstrated a sensitivity of 38.8% and a specificity of 96.7% when low-risk neoplasms were classified as benign, with an overall accuracy of 78.2%. When low-risk neoplasms were classified as malignant, sensitivity decreased to 31.4%, while specificity remained 96.7%, with an overall accuracy of 70.4%. In the Bethesda III–IV subset (*n* = 153), the corresponding values were 24.5% sensitivity and 95.2% specificity under the benign classification, and 20.0% sensitivity with 95.5% specificity under the malignant classification. Overall accuracy in this subset was 72.5% and 63.4%, respectively (Table 4).

Among the 124 patients who were operated on and whose nodules were found to be malignant, mutation status was analyzed in relation to clinicopathological features. In terms of tumor size (T), a statistically significant difference was observed across groups (*p* = 0.028). Post hoc pairwise analysis demonstrated that mutation positivity was higher in T1b (58%) and T3a (56.3%) tumors, whereas it was significantly lower in T2 tumors (25%). Lymph node status (N), metastasis (M), and TNM staging did not show significant differences by mutation status. Mutation positivity was similar across ATA risk groups and levels of extrathyroidal and vascular invasion, with no statistically significant associations observed for these parameters (Table 5).

## 4. Discussion

Fine-needle aspiration cytology (FNAC) remains the preferred method for differentiating benign from malignant thyroid nodules. However, indeterminate cytological categories (TBSRTC III–V) present substantial clinical challenges. Nonetheless, accumulating evidence suggests that this limitation can be addressed through molecular diagnostic approaches based on comprehensive genomic analyses [8]. Therefore, identifying biomarkers with high specificity and sensitivity to enable the development of rapid, cost-effective, and reliable diagnostic techniques remains a critical clinical priority.

To our knowledge, no previous research has concurrently evaluated both BRAF and RAS mutations using this approach [9]. Therefore, our study not only contributes to the growing literature on minimally invasive molecular diagnostics but also provides a novel approach to the combined assessment of these two key oncogenic drivers, which can be studied in fresh tissue samples at low cost, which is crucial. Another major objective of our research was to assess the feasibility and success rate of DNA extraction from FNA washout fluid, which remains a technically challenging yet potentially valuable source for molecular analysis. Importantly, our institution is a tertiary referral center for the diagnosis and management of thyroid cancer, and our relatively large sample size compared to previously published studies strengthens the reliability and generalizability of our findings.

Among those recommended for surgery, 5.6% declined the operation and were subsequently lost to follow-up, emphasizing the importance of patient preference and long-term monitoring in management outcomes. Notably, histopathological results of the operated group revealed that nearly 60% of nodules were benign and 40% malignant. In a recent meta-analysis conducted by the Italian Consensus for the Classification and Reporting of Thyroid Cytology (ICCRTC) group, the overall operation rate for Bethesda category III and IV nodules was reported as 54.3%. When analyzed separately, the operation rate was approximately 45% for Bethesda III and 75% for Bethesda IV nodules, and the malignancy rate was 40%, figures closely aligning with the proportions observed in our cohort, further supporting the consistency of surgical decision-making patterns across different populations and study settings [10].

In our study, molecular analysis from FNA washout material was technically feasible in the vast majority of cases, demonstrating a high adequacy rate for DNA extraction and testing (90%), confirming the practicality of this approach in routine cytology workflows. Mutation positivity was identified in a modest proportion of patients, while the majority exhibited wild-type results, consistent with the expected prevalence of these mutations in unselected indeterminate thyroid nodules. Similarly, in a previous report, in unselected indeterminate thyroid nodules of 115 cases, FNA washout precipitation specimens were effective supplements to the thyroid genetic tests in 100% of patients [11].

In our cohort, NRAS mutations, particularly those involving codon 61, were the most frequent molecular alterations, followed by BRAF V600E and its complex variants, while KRAS mutations were rare, which was compatible with previous reports [12]. This distribution aligns with the known mutational landscape of indeterminate thyroid nodules, in which RAS-type mutations predominate in follicular-patterned lesions, whereas BRAF V600E is typically associated with papillary carcinoma morphology [13].

In line with the WHO 2022 classification, our findings demonstrated that papillary thyroid carcinoma (PTC) was the predominant histologic subtype among malignant and borderline tumors, showing a clear association with mutation positivity. This observation supports the well-established link between BRAF and RAS pathway alterations and papillary-type morphology [14]. More than half of the malignant cases in our cohort harbored either BRAF or RAS mutations, consistent with prior studies reporting a similar overall mutation prevalence. In one such study, 52% of thyroid tumor specimens carried clinically relevant mutations in at least one screened gene, most frequently involving KRAS (86%), followed by NRAS (7%), BRAF (6%), and combined NRAS + BRAF mutations (2%) [15].

In our study, the combined sensitivity and specificity of mutation testing were 48% and 89.6%, respectively, with a positive predictive value (PPV) of 75.9%, a negative predictive value (NPV) of 71.9%, and an overall diagnostic accuracy of 72.9%. The slight variation in performance depending on whether borderline lesions were classified as benign or malignant reflects the intrinsic biological ambiguity of these entities. Nevertheless, the relatively high specificity and PPV indicate that the detection of a BRAF or RAS mutation substantially increases the likelihood of malignancy. Similarly, in a previous study using a PCR-based method, BRAF mutations were detected in 17.1% (13/76) of cases, yielding 16.7% sensitivity, 100% specificity, 100% PPV, and 82.8% NPV in the AUS category, and 73.3% sensitivity, 100% specificity, 100% PPV, and 20% NPV in the Bethesda V group [16]. For RAS mutations alone, the diagnostic value for malignancy detection was relatively low in our study, concordant with the literature. This finding is consistent with previous data reporting modest predictive performance for RAS positivity in indeterminate thyroid nodules. In an earlier meta-analysis, the weighted averages for positive and negative predictive values were 78.0% and 64.0%, respectively, with an overall diagnostic accuracy of 68.0%. The corresponding positive likelihood ratio was 4.235 (95% CI, 1.506–11.910), and the negative likelihood ratio was 0.775, underscoring the limited standalone utility of RAS testing and the importance of interpreting such results in conjunction with cytologic and clinical findings [17].

Lymph node involvement, distant metastasis, and overall TNM stage did not differ significantly by mutation status, indicating that BRAF and RAS mutations alone are not reliable predictors of tumor extent or aggressiveness. The literature presents conflicting evidence regarding the prognostic impact of these mutations. A meta-analysis including 2552 thyroid cancer patients from 17 studies reported an overall RAS mutation prevalence of 35.4% (95% CI: 22.7–50.7%), with NRAS being the most frequent subtype (69.5%), followed by HRAS (25.8%) and KRAS (6.9%). No significant differences were observed between RAS-positive and RAS-negative cases in early tumor stage (T1/T2), lymph node metastasis, extrathyroidal extension, or recurrence rates. However, distant metastasis was significantly more common in RAS-positive tumors (15%, 95% confidence interval (CI): 6–34%) compared with RAS-negative ones (4%, 95% CI: 1–12%), corresponding to a relative risk of 3.23 (95% CI: 1.49–7.02) [18]. Regarding BRAF, its prevalence ranges from 46% to 90% among papillary thyroid carcinomas (PTCs) identified on cytology. While higher-risk tumors more frequently harbor BRAFV600E mutations, this alteration is also detected at a high rate in low-risk PTCs. Some evidence suggests that a higher allelic frequency of the BRAFV600E mutation may be associated with more aggressive tumor behavior, though the exact prognostic significance remains under debate [19].

This study has several limitations. First, the proportion of tumor cells within fresh thyroid FNA samples was not determined. Consequently, some specimens may have contained few or no tumor cells, potentially falling below the detection threshold of the molecular assay. Second, mutation validation was not conducted on histopathological specimens.

## 5. Conclusions

Our study demonstrated that BRAF and RAS mutation analysis performed on fresh FNA washout material is a feasible, accurate, and cost-effective adjunct for evaluating indeterminate thyroid nodules. Molecular testing was successfully achieved in more than 90% of the samples, with high concordance between washout and tissue-based results, confirming the reliability of this approach in routine practice. NRAS codon 61 mutations were the most common alterations, followed by BRAF V600E, consistent with known molecular patterns in follicular-derived neoplasms. Mutation positivity yielded a combined sensitivity of 48%, a specificity of 89.6%, and a diagnostic accuracy of 72.9%, significantly improving malignancy prediction. Importantly, 40% of surgically resected nodules were malignant—closely matching prior large-scale meta-analyses—further supporting the clinical relevance of our findings. Overall, these results highlight that combined BRAF and RAS testing from FNA washout specimens represents a practical and informative tool to enhance diagnostic precision and optimize management decisions in patients with indeterminate thyroid cytology.

## Figures and Tables

**Table 1:** Sample material adequacy and mutation status in thyroid FNA washout specimens.

Sample Material Adequacy	Count (n)	Percentage (%)
Sufficient material obtained (Yes)	1037	90.9
Not sufficient (No)	12	1.1
Partially sufficient for BRAF	9	0.8
Partially sufficient for RAS	27	2.4
Sufficient except BRAF V600E	2	0.2
Sufficient except KRAS	16	1.4
Sufficient except NRAS	30	2.6
Not analyzed	6	0.5
Total	1139	100.0

**Table 2:** Distribution of BRAF, NRAS, and KRAS Mutations in the overall cohort and operated subset.

Detected Mutation	Overall Cohort (*n* = 1139, % of Positives)	Surgically Treated Subgroup (*n* = 307, % of Positives)
**BRAF mutations**	**55 (43.0%)**	**44 (55.7%)**
V600E (c.1799T>A)	11 (8.6%)	8 (10.1%)
V600E + V600E complex (c.1799_1880delinsAA)	43 (33.6%)	35 (44.3%)
V600E complex only (c.1799_1880delinsAA)	1 (0.8%)	1 (1.3%)
**NRAS mutations**	**67 (52.3%)**	**32 (40.5%)**
Q61K/R/L (c.181C>A, c.182A>G, c.182A>T)	49 (38.3%)	18 (22.8%)
NRAS Q61R (c.182A>G)	10 (7.8%)	7 (8.9%)
NRAS Q61K/L/R/H (c.181C>A, c.182A>T, c.182A>G, c.183A>C/T)	6 (4.7%)	5 (6.3%)
NRAS C12S/C–G12A/D/V–G13R/D/V	1 (0.8%)	1 (1.3%)
**KRAS mutations**	**5 (3.9%)**	**2 (2.5%)**
KRAS G12V/D/A (suspicious positive c.35G>T/A/C)	1 (0.8%)	1 (1.3%)
KRAS G13D (c.38G>A)	2 (1.6%)	1 (1.3%)
KRAS A59T/E/G (c.175G>A, c.176C>A/C>G)	1 (0.8%)	–
KRAS A146T/V (suspicious positive c.436G>A, c.437C>T)	1 (0.8%)	–
**BRAF + NRAS co-mutation** (V600E + V600E complex + NRAS Q61K/R/L)	**1 (0.8%)**	**1 (1.3%)**
**Total mutation-positive cases**	**128 (11.2%)**	**79 (25.7%)**

**Table 3:** Diagnostic performance of BRAF or RAS mutation status according to low-risk neoplasm classification in the overall and Bethesda III–IV cohorts.

All Cases (*n* = 307)	Mutation Status	Cancer (+)	Cancer (−)	Total	Sens	Spec	Acc	PPV	NPV
Low-risk neoplasms	Positive	50	29	79	51.0	86.1	74.0	63.3	78.9
considered benign	Negative	48	180	228					
Low-risk neoplasms	Positive	60	19	79	48.0	89.6	72.9	75.9	71.9
considered malignant	Negative	64	164	228					
Bethesda III–IV Subset (*n* = 153)									
Low-risk neoplasms	Positive	19	21	40	38.8	79.8	66.6	47.5	73.5
considered benign	Negative	30	83	113					
Low-risk neoplasms	Positive	19	21	40	41.0	85.2	66.0	67.5	66.4
considered malignant	Negative	30	83	113					

**Table 4:** Diagnostic performance of isolated BRAF positivity according to low-risk neoplasm classification in the overall and Bethesda III–IV Cohorts.

All Cases (*n* = 307)	Mutation Status	Cancer (+)	Cancer (−)	Total	Sens	Spec	Acc	PPV	NPV
Low-risk neoplasms	Positive	38	7	45	38.8	96.7	78.2	84.4	77.1
considered benign	Negative	60	202	262					
Low-risk neoplasms	Positive	39	6	45	31.4	96.7	70.4	86.7	67.6
considered malignant	Negative	85	177	262					
Bethesda III–IV Subset (n = 153)									
Low-risk neoplasms	Positive	12	5	17	24.5	95.2	72.5	70.6	72.8
considered benign	Negative	37	99	136					
Low-risk neoplasms	Positive	13	4	17	20.0	95.5	63.4	76.5	61.8
considered malignant	Negative	52	84	136					

**Table 5:** Comparison of clinicopathological features by mutation status.

Parameter	Category	Mutation (−) *n* = 64	Mutation (+) *n* = 60	Mutation (+) (%)	*p* Value
**T (Primary tumor)**	TX	0	1	100.0%	
	T1a ≤ 1 cm	14	14	50.0%	
	T1b > 1–2 cm	21	30	58.8%	
	T2 > 2–4 cm	21	6	22.2%	
	T3a > 4 cm	8	9	52.9%	**0.028**
**N (Lymph node)**	NX	3	0	0.0%	
	N0a	18	25	58.1%	
	N0b	38	29	43.3%	
	N1a	5	6	54.5%	0.150
**M (Metastasis)**	M0	64	60	48.4%	—
**TNM Stage (≥55 yrs)**	Stage I	60	55	47.8%	
	Stage II	3	5	62.5%	
	Stage IVB	1	0	0.0%	0.452
**ATA Risk Class.**	Low risk	41	39	48.7%	
	Intermediate risk	21	19	47.5%	
	High risk	2	2	50.0%	0.990
**Extrathyroidal invasion**	None	49	43	46.7%	
	Minimal	7	11	61.1%	
	Muscle invasion	1	0	0.0%	0.333
**Vascular invasion**	None	44	41	48.2%	
	Focal	6	4	40.0%	
	Indeterminate	15	12	44.4%	
	Suspicious	3	1	25.0%	0.789

## Data Availability

The data are not publicly available due to privacy and ethical restrictions.

## Abbreviations

AUS	Atypia of Undetermined Significance
ATA	American Thyroid Association
CI	Confidence Interval
CT	Cycle Threshold
FN	Follicular Neoplasm
FNA	Fine-Needle Aspiration
FNAB	Fine-Needle Aspiration Biopsy
FNAC	Fine-Needle Aspiration Cytology
NIFTP	Noninvasive Follicular Thyroid Neoplasm with Papillary-like Nuclear Features
NPV	Negative Predictive Value
PPV	Positive Predictive Value
PTC	Papillary Thyroid Carcinoma
RT-PCR	Real-Time Polymerase Chain Reaction
SD	Standard Deviation
TBSRTC	The Bethesda System for Reporting Thyroid Cytopathology
TNM	Tumor–Node–Metastasis
UMP	Tumor of Uncertain Malignant Potential
WHO	World Health Organization
